# Supplementary material for: Rab46 integrates Ca2+ and histamine signaling to regulate selective cargo release from Weibel-Palade bodies
Source: J Cell Biol. 2019 May 15;218(7):2232–46. doi: 10.1083/jcb.201810118 (PMC6605797; doi:10.1083/jcb.201810118)
Supplement: Supplemental Materials (PDF) [file JCB_201810118_sm.pdf]

JCB

Supplemental material

Miteva et al., https://doi.org/10.1083/jcb.201810118

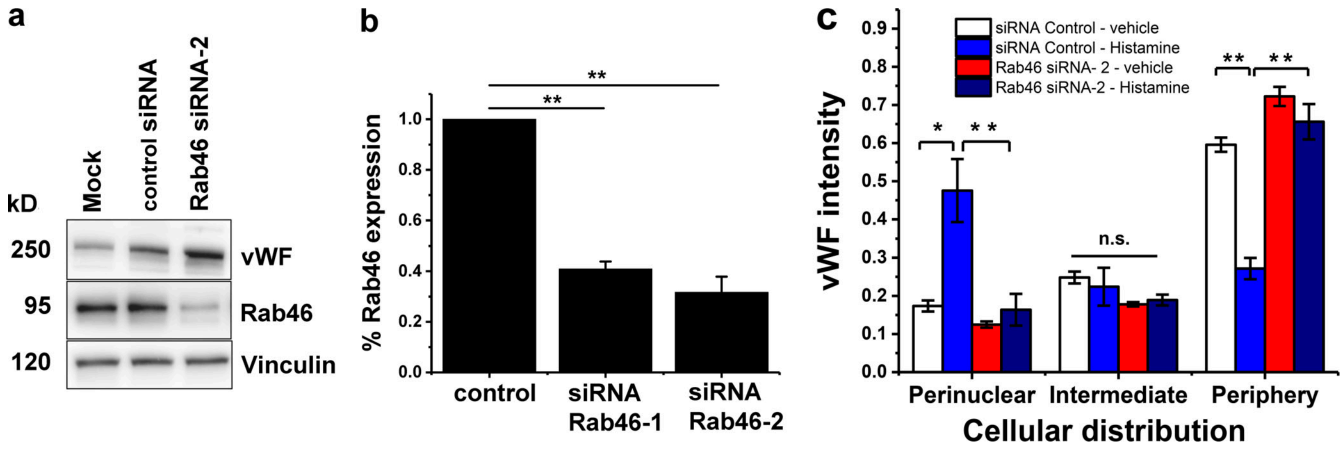

Figure S1. **Rab46 depletion using targeted siRNAs. (a and b)** Representative Western blot (a) and mean data (b) of HUVEC lysate (transfected with control siRNA and two siRNAs targeting different regions of Rab46: siRNA Rab46-1 and siRNA Rab46-2), confirming reduced expression of Rab46 protein 48 h after transfection. The graph shows mean ± SEM of densitometry analysis of relative band intensity normalized to vinculin. *n* = 6; **, P < 0.01. **(c)** vWF cellular distribution in stimulated HUVECs transfected with Rab46 siRNA-2 compared with control siRNA is the same as HUVECs transfected with siRNA-1. In control conditions (vehicle) or after 10-min histamine treatment (30 µM), cells were imaged, and vWF intensity was quantified. The graph shows the quantitative analysis of vWF distribution in scrambled control cells and Rab46-depleted cells upon histamine stimulation. Results were grouped into three areas: perinuclear, intermediate, and periphery. The plot shows vWF signal intensity of each particle in the respective area, where the mean (± SEM) was noted as a percentage of the total signal intensity. Number of independent biological repeats/technical repeats = 3/15–18. **, P < 0.01.

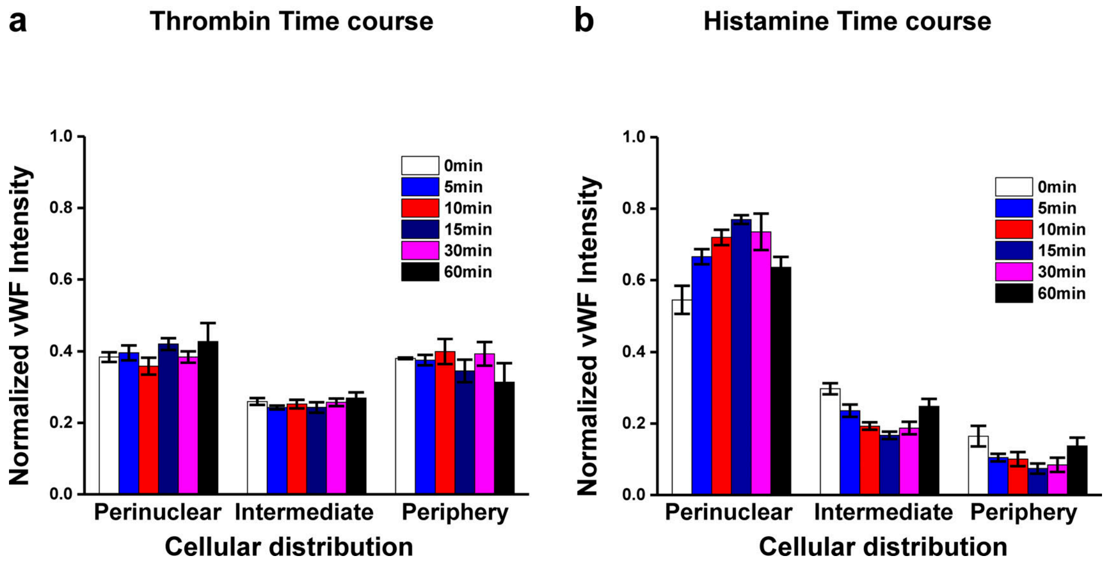

Figure S2. **Histamine but not thrombin evokes time-dependent perinuclear trafficking of WPBs. (a and b)** vWF cellular distribution in response to 2.5 U/ml thrombin (a) and 30 µM histamine (b) treatment for 0, 5, 10, 15, 30, and 60 min. The plot represents mean ± SEM normalized vWF intensity in the respective area. Number of independent biological repeats/technical repeats = 3/18–30.

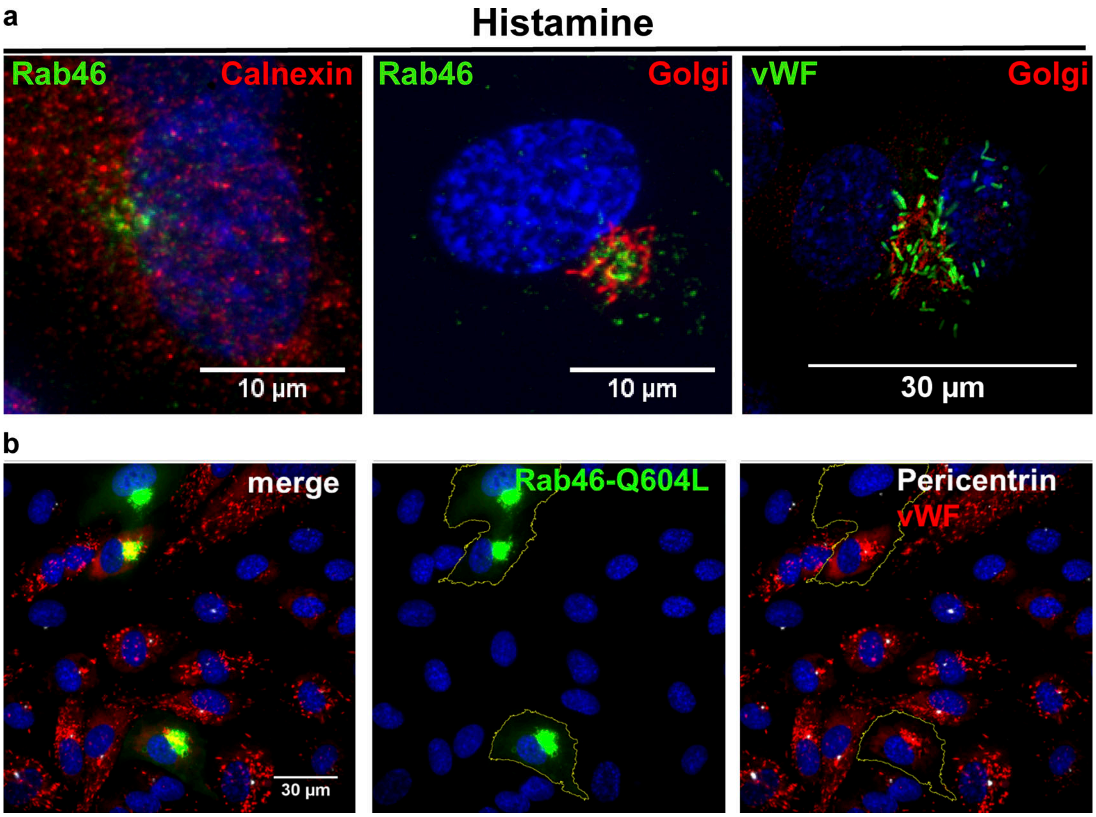

Figure S3. **Histamine stimulation evokes localization of Rab46 at the MTOC. (a)** Representative DeltaVision image of HUVECs treated with 30 µM histamine and costained with Rab46 (green) and calnexin (red) as an ER marker (left). Representative image of HUVECs costained with Rab46 (green) and GM130 (red) as Golgi marker (middle) and Airyscan image of vWF (green) and anti 58K (red: right). Number of independent biological repeats/technical repeats = 3/10. **(b)** Constitutively active (Q604L) Rab46 localizes vWF to the MTOC in the absence of stimulation. DeltaVision images of HUVECs immunostained for Rab46 Q604L (green), vWF (red), and pericentrin (white) as a marker of the MTOC in control conditions. vWF localizes to the perinucleus in cells expressing Q604L in the absence of stimulation. Scale bar = 30 µm.

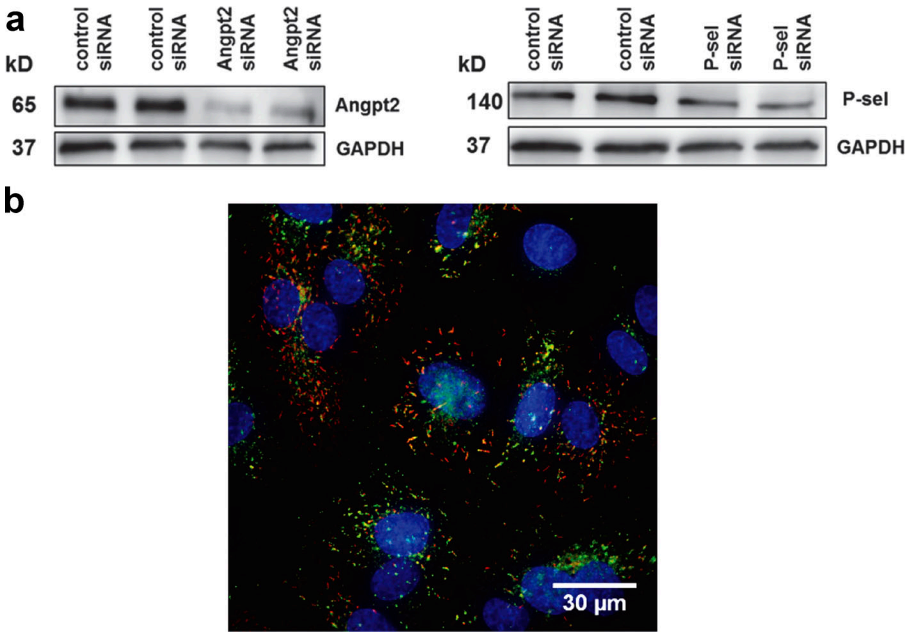

Figure S4. **Example Western blot analysis depicting the specificity of the anti-angpt2 and anti–P-selectin antibodies. (a)** Anti-angpt2 and anti–P-selectin antibodies recognize their respective protein bands in Western blots and reduced-intensity bands when cells have been transfected with control siRNA or siRNA targeted against angpt2 (left) or P-selectin (right). *n* = 3. **(b)** 0.3 µM histamine does not induce clustering of Rab46. Representative image of HUVECs stimulated with 0.3 µM histamine for 10 min and stained for endogenous Rab46 (green) and vWF (red). Scale bar = 30 µm.

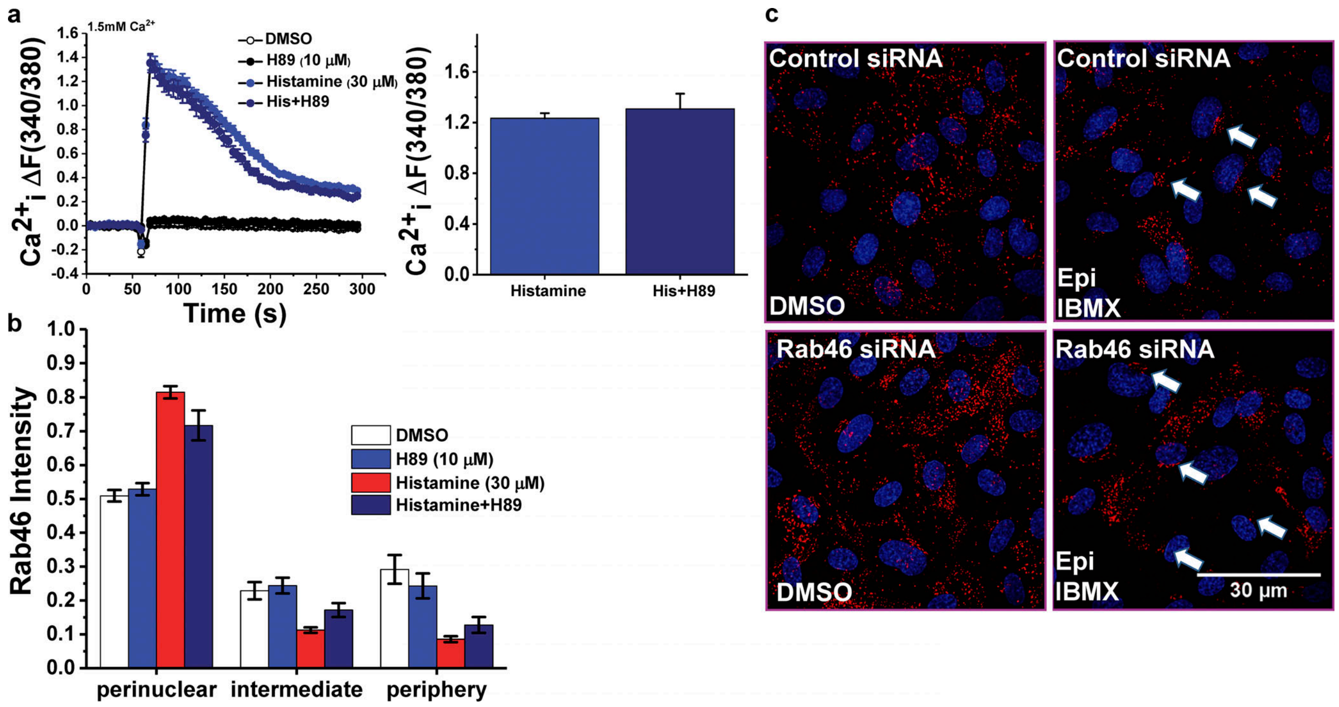

Figure S5. **Histamine-evoked perinuclear clustering is cAMP independent, and Rab46 is not necessary for cAMP-dependent clustering. (a)** Representative traces and mean data of the calcium response evoked by histamine in HUVECs in the presence or absence of the PKA inhibitor H-89. Representative traces of change (Δ) in intracellular Ca^{2+} evoked by histamine in HUVECs loaded with the Ca^{2+} indicator Fura-2-AM. Number of independent biological repeats/technical repeats = 3/18. **(b)** Mean data showing the cellular distribution of Rab46 upon histamine stimulation in cells pretreated with H-89 or respective control. The plots quantify Rab46 signal intensity in the respective area, where the mean (± SEM) was noted as a percentage of the total signal intensity. Number of independent biological repeats/technical repeats = 3/18; *, $P < 0.05$. **(c)** vWF (red) clusters in HUVECs in response to 15-min treatment with 100 µM epinephrine plus 100 µM IBMX (arrows: top right) compared with control (top left). vWF clusters in response to epinephrine plus IBMX in cells transfected with Rab46 siRNA-1 (arrows: bottom right). *n* = 3. Scale bar = 30 µm.

Table S1. **List of primers for CRACR2A-L (Rab46) mutagenesis**

Mutation	Primer (5′–3′)
Rab46_T559N	
Forward	GGTGGGGAAGAATTCCTTCCTGAGG
Reverse	CCTCAGGAAGGAATTCTTCCCCACC
Rab46_Q604L	
Forward	TGGGACACGGCTGGGCTGGAGAGGTACCGGTGC
Reverse	GCACCGGTACCTCTCCAGCCCAAGCCGTGTCCCA
Rab46_N559I	
Forward	TGTTCTTCTGCTGGGTATTAAGCTTGACAACGAG
Reverse	CTCGTTGTCAAGCTTAATACCCAGCAGAAGAACA
Rab46_EFmutant (+E108Q)	
Forward	GTGTTTGATGCCCTGGCTGCTGCTGGCAATGGCTATCTG
Reverse	CAGATAGCCATTGCCAGCAGCAGCCAGGGCATCAAACAC
Forward	TATCTGACCCCACAGCAGTTCACTACTGGATTT
Reverse	AAATCCAGTAGTGAACTGCTGTGGGGTCAGATA

https://doi.org/10.1083/jcb.201810118

Table S2. **Primary antibodies used for immunocytochemistry and Western blotting**

Primary antibody	Species	Working dilution	Supplier
Anti-human vWF	Mouse	1:200	Dako
Anti-human EFCAB4B	Rabbit	1:100	Proteintech
Anti-58K Golgi protein	Mouse	1:100	Genetec
Anti-pericentrin	Mouse	1:100	Abcam
Anti-pericentrin	Rabbit	1:1,000	Abcam
Anti-Rab11	Mouse	1:100	Santa Cruz
Anti-Lamp1	Mouse	1:100	Developmental Studies Hybridoma Bank
Anti-tpA	Mouse	1:100	Santa Cruz
Anti–P-selectin	Mouse	1:100	Santa Cruz
Anti-DYNC1H1	Rabbit	1:1,000	Proteintech
Anti–angpt2	Goat	1:100	R&D Biosystems

Table S3. **Secondary antibodies used for immunocytochemistry and Western blotting**

Secondary antibody	Dilution	Supplier
Alexa Fluor 488 anti-rabbit IgG	1:300	Jackson ImmunoResearch Labs
Alexa Fluor 594 anti-mouse IgG	1:300	Jackson ImmunoResearch Labs
Alexa Fluor 647 anti-mouse IgG	1:300	Jackson ImmunoResearch Labs
Alexa Fluor 594 anti-goat IgG	1:300	Jackson ImmunoResearch Labs
HRP anti-mouse IgG	1:5,000	Jackson ImmunoResearch Labs
HRP anti-mouse IgG	1:10,000	Jackson ImmunoResearch Labs

Table S4. **siRNA sequences**

siRNA target	Sequence (5′–3′)	Supplier and catalog number
siRNA #1 EFCAB4B	GUGUGAAGGUCAAAAGAGAtt	Ambion 4390771
siRNA #2 EFCAB4B	GGAGUUCACUACUGGAUUUtt	Ambion 4392420
siRNA angpt2	CCUUCCAACUUGAACGGAAtt	Ambion 4392420
siRNA P-selectin (4× mixed pool)	GCUGAGAGGAGCCGAUAUA	Dharmacon L-008079-00-0005
	GCUGAGAACUGGGCUGAUA	Dharmacon L-008079-00-0005
	GUAAAGCUGUGCAGUGUCA	Dharmacon L-008079-00-0005
	CUAGAGGGCCAGUUACUUA	Dharmacon L-008079-00-0005
Control siRNA	UGGUUUACAUGUCGACUAA	Dharmacon D-001810-01-05 #1
Control siRNA	Unknown	Ambion Silencer negative control no. 1 a.m.4611

Table S5. **RT-PCR primers**

Target	Primer (5′–3′)
vWF	
Forward	TTCCCGACAAGGTGTGTGTC
Reverse	GCCTTCATGCAGAACGTAAGTG
Rab46	
Forward	GGTCATCCTTGCCTACG
Reverse	GCTCGCATGAGATCAAGT
Angpt2	
Forward	CAAATGCTAACAGGAGGCTGGT
Reverse	CAGGTGGACTGGGATGTTTAGA
H1R	
Forward	TCTCTCTTTTCTGTGGGTTATTCC
Reverse	CAGCTTAATTTCTGAGAAGGAAGG
H4R	
Forward	TGATCCCAGTCATCTTAGTCGCTTATTTC
Reverse	GGAGAAGGAACCCATTTTGGAAGCAA

Provided online is Data S1, an ImageJ macro used for analyzing the cellular distribution of Rab46 and vWF.
